# QSPR model for Caco-2 cell permeability prediction using a combination of HQPSO and dual-RBF neural network†

**DOI:** 10.1039/d0ra08209k

**Published:** 2020-11-26

**Authors:** Yukun Wang, Xuebo Chen

**Affiliations:** School of Chemical Engineering, University of Science and Technology Liaoning No. 185, Qianshan Anshan 114051 Liaoning China wyk410@163.com; School of Electronic and Information Engineering, University of Science and Technology Liaoning No. 185, Qianshan Anshan 114051 Liaoning China xuebochen@126.com +864125928367

## Abstract

The Caco-2 cell model is widely used to evaluate the *in vitro* human intestinal permeability of drugs due to its morphological and functional similarity to human enterocytes. Although it is safe and relatively economic, it is time-consuming. A rapid and accurate quantitative structure-property relationship (QSPR) model of Caco-2 permeability is helpful to improve the efficiency of oral drug development. The aim of our study is to explore the predictive ability of the QSPR model, to study its permeation mechanism, and to develop a potential permeability prediction model, for Caco-2 cells. In our study, a relatively large data set was collected and the abnormal data were eliminated using the Monte Carlo regression and hybrid quantum particle swarm optimization (HQPSO) algorithm. Then, the remaining 1827 compounds were used to establish QSPR models. To generate multiple chemically diverse training and test sets, we used a combination of principal component analysis (PCA) and self-organizing mapping (SOM) neural networks to split the modeling data set characterized by PaDEL-descriptors. After preliminary selection of descriptors by the mean decrease impurity (MDI) method, the HQPSO algorithm was used to select the key descriptors. Six different methods, namely, multivariate linear regression (MLR), support vector machine regression (SVR), xgboost, radial basis function (RBF) neural networks, dual-SVR and dual-RBF were employed to develop QSPR models. The best dual-RBF model was obtained finally with *R*^2^ = 0.91, and *R*_cv5_^2^ = 0.77, for the training set, and *R*_T_^2^ = 0.77, for the test set. A series of validation methods were used to assess the robustness and predictive ability of the dual-RBF model under OECD principles. A new application domain (AD) definition method based on the descriptor importance-weighted and distance-based (IWD) method was proposed, and the outliers were analyzed carefully. Combined with the importance of the descriptors used in the dual-RBF model, we concluded that the “H E-state” and hydrogen bonds are important factors affecting the permeability of drugs passing through the Caco-2 cell. Compared with the reported studies, our method exhibits certain advantages in data size, transparency of modeling process and prediction accuracy to some extent, and is a promising tool for virtual screening in the early stage of drug development.

## Abstract 2

The aim of this study is to establish a promising QSPR model for the Caco-2 permeability prediction.

## Graphical abstract

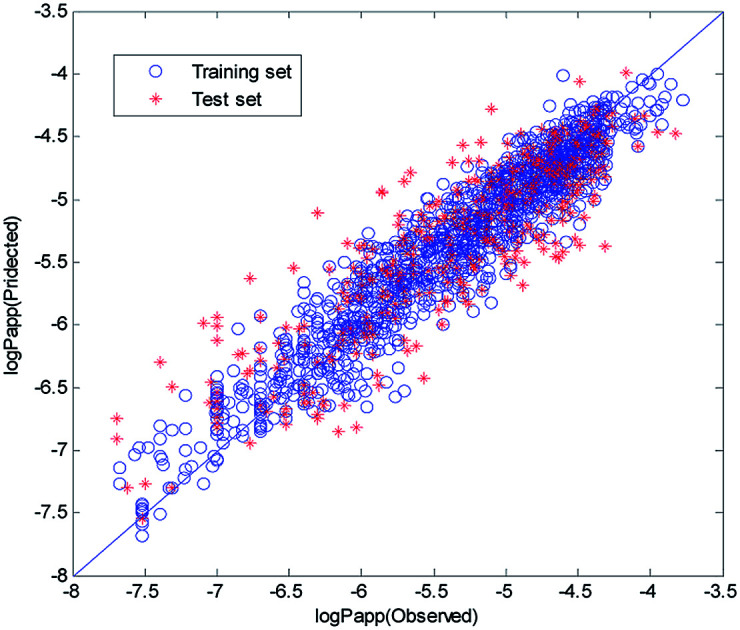

## 1. Introduction

In the process of drug development, lot of candidate drugs fail to become drugs mainly because of their safety issues and lack of efficacy.^1^ This is also the main reason for high costs and time-consumption in pharmaceutical engineering. In every stage of drug discovery and development, absorption, distribution, metabolism, excretion, and toxicity (ADMET) properties of chemicals play vital role; so rapid evaluation of ADMET is the key to improve the efficiency of drug development.^2^ For a new oral drug, bioavailability, reflecting the drug proportion in the circulatory system, is a significant index of the drug efficacy. Screening for absorption ability is one of the most important part of assessing oral bioavailability.^3^ The small intestine is the major absorption site of oral drugs, so poor intestinal absorption is prone to cause higher probabilities of failure in the early stage of drug discovery.^4^ Therefore, the evaluation of absorption ability for oral drugs is crucial in ADMET profiling. Reported studies have demonstrated that there was an apparent correlation between the human intestinal absorption and its intestinal permeability for a drug.^5–8^ We can evaluate the intestinal absorption capacity of drugs by their intestinal permeability. The Caco-2 cell model is widely used to evaluate the *in vitro* human intestinal permeability of drugs due to its morphological and functional similarity to human enterocytes. However, it is difficult to accomplish high-throughput screening (HTS) with the traditional Caco-2 cell model due to its long culturing period (21 days) allowing for full cell differentiation into the enterocyte-like phenotype.^9^ Moreover, the 21 days culture necessary for Caco-2 cells not only increases the probability of contamination but also brings undesirable high costs for drug discovery.^10^ Although the scientists have managed to shorten the culturing period of Caco-2 cells to seven days by various efforts,^4^ the traditional experimental methods are still expensive and time-consuming. It is difficult to realize the HTS of drugs, not to mention the virtual screening in the early stage of drug discovery.^3,11^ Therefore, a rapid, accurate and economic model of Caco-2 permeability is the key to improve the efficiency of oral drug development.

QSPR models are ideal alternatives and have been widely applied to Caco-2 permeability prediction due to their higher efficiency and lower cost. At present, the published QSPR models can mainly be divided into two types: regression models and classification models. Now, there are fewer classification models than regression models in predicting Caco-2 cell permeability. The main reason is that the accuracy of classification model is limited, which will lead to wrong classification.^12–14^ Compared with the regression model, it is easier to delete the promising candidate drugs. Therefore, in our study, we focus on regression model.

As far as we know, the reported regression models for Caco-2 cell permeability were quite different in terms of data size, descriptor type and quantity, and modeling method. In terms of data size, the smallest model consists of only 17 molecules,^5^ and the largest model consists of 15 791 molecules.^15^ In terms of descriptor type and quantity, there were some kinds of descriptors such as MOE descriptors,^3,15^ Molconn-Z descriptors,^16^ and E-Dragon descriptors^17^ used in these models, including 2D and 3D descriptors, and the number of selected descriptors ranges from 4 to 70. In terms of modeling methods, linear modeling methods such as multivariate linear regression (MLR),^3,18,19^ and nonlinear modeling methods such as partial least-squares (PLS),^3,5,20^ KNN,^21^ support vector machine (SVM),^3,22^ random forest (RF)^15^ and boosting^3^ were used to construct these QSAR/QSPR models.

Although these Caco-2 permeability prediction models have relatively reasonable accuracy, they still have some shortcomings, mainly in the following aspects: (1) some models used very little modeling data,^5,16,22,23^ even less than 100 compounds. Although they have relatively high prediction accuracy, it is difficult to collect sufficient and diverse molecular information for them to develop a QSPR model with superior performance and a wide application domain (AD). (2) Some models used 3D descriptors,^3,16,19,23^ which may be beneficial to improve the accuracy and mechanism interpretation. However, when calculating 3D descriptors, molecular structure optimization is inevitable. The complexity of the molecular structure and the limitations of existing optimization algorithms may lead to time-consumption and unstable results.^24^ This will bring instability to the models and limit their rapid application. (3) The modeling process of some models was not standardized and did not comply with the Organization for Economic Co-operation and Development (OECD) principles. Some models lacked test sets or had a small test set,^5,16,22,23^ some models were not cross-verified,^18,21,23^ some models lacked AD,^16,17,19,23^ and some models lacked mechanistic interpretation.^16,23^ In addition, the modeling process of some models^3,11,23^ is not transparent enough, which leads to poor repeatability and reproducibility. These shortcomings limit their usefulness as an effective drug screening tool. The aim of our study is to try to overcome these shortcomings and establish a standardized and efficient QSPR model with better fitting ability, robustness, and external predictive ability, as well as a wide AD. Meanwhile, the mechanism of permeability and causes of outliers should be explained reasonably. To achieve this goal, a relatively large and chemically diverse Caco-2 permeability data set characterized by PaDEL-descriptors was used to establish our Caco-2 permeability prediction models.

Primarily, we cleaned the abnormal data in the original data set, obtained the modeling data set containing 1827 molecules, and selected 50 key descriptors for QSPR models using the multivariate linear regression (MLR) and hybrid quantum particle swarm optimization (HQPSO) algorithm. Subsequently, six different methods, namely, multivariate linear regression (MLR), support vector machine regression (SVR), xgboost, radial basis function (RBF) neural networks, dual-SVR and dual-RBF were employed to develop QSPR models and the best one was chosen for further analyses.

According to the principle of OECD, a variety of validation methods were used to evaluate the robustness and prediction ability of the best dual-RBF model, and then we evaluated the importance of the descriptors by the mean decrease impurity (MDI) method^25^ and concluded that “H E-state” and hydrogen bond are important factors affecting the permeability of compounds passing through Caco-2 cells. Finally, a new AD definition method with a descriptor importance-weighted and distance-based (IWD) method was proposed to define its AD. Compared with previous published Caco-2 permeability QSAR/QSPR models, our new dual-RBF QSPR model is able to make up the existing disadvantages to some extent. The results indicate that the proposed model is normative, transparent and robust. It can predict the permeability values of new compounds quickly and reliably, and hence, it can be developed into a promising drug screening tool.

## 2. Experimental section

### 2.1. Modeling overview

**Fig. 1:**
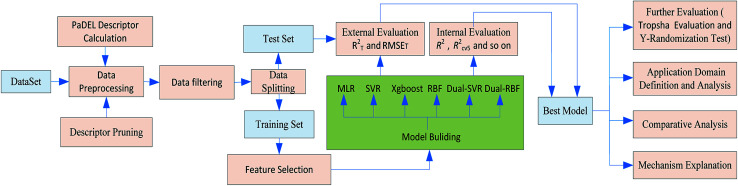
Flowchart of the modelling process for Caco-2 permeability QSPR models.

A workflow for the modeling process is shown in Fig. 1. Specific details of each step are provided in subsequent sections.

### 2.2. Data collection and descriptor pruning

It is well known that more the number of chemically diverse compounds used for QSAR modeling, the wider is the AD of the obtained model. To our knowledge, the largest QSAR model consists of 15 791 compounds.^15^ Unfortunately, the authors did not provide detailed information of compounds used in their studies. In our study, a relatively large Caco-2 permeability data set was collected partly from the literature^3^ and partly from the CHEMBL data set (https://www.ebi.ac.uk/chembl), including aldehyde, carboxylic acid, esters, amines, alkanes, alkenes, alkynes, alcohols, nitrobenzene, halohydrocarbons, and ketones. These data were chaotic due to their different sources. To improve the quality and reliability of the data, we coped with them as follows:

(1) Deleted compounds that do not have a clear Papp value and compounds without clear SMILES code.

(2) If two or more compounds have the same SMILES, when their Papp values were not significantly different, we took their arithmetic mean as the final Papp value. When their Papp values differ greatly, we deleted them to avoid errors.

(3) The data set was filtered to remove compounds with Papp values greater than 10^−3.5^ cm s^−1^ or less than 10^−8^ cm s^−1^ because of their potential unreliability.^3^

(4) To compress the scale of variables and eliminate heteroscedasticity, we selected log Papp as the endpoint of these data.^3,17^

To construct a stable QSAR model, we only calculated 0–2D PaDEL descriptors to avoid uncertainty caused by molecular structure optimization when calculating 3D descriptors. The descriptors were calculated through online website (http://www.scbdd.com/padel_desc/index). After that, we examined each compound carefully. If a compound had a null descriptor and the variance of this descriptor in other compounds was greater than 0.3, then we removed that compound to avoid deleting important descriptors in subsequent steps.

To simplify the model structure and reduce redundancy, two pretreatments were performed to delete some uninformative or redundant descriptors before further selection:

(1) Descriptors with constant or null values were excluded.

(2) If the descriptors were found to be correlated pairwise (greater than 0.85), then the descriptor that had the least correlation with a Caco-2 permeability value (log Papp) was excluded.

Finally, the initial modeling data set with 1864 compounds and 261 descriptors was reserved. The data set was listed in the Supplementary Materials (Initia_modeling_data.xls).†

### 2.3. Data filtering

The crucial step of building a high-performance QSAR/QSPR model is the filtering of abnormal data in the model.^26^ Young *et al.* studied 6 public and private databases, and found that the ratio of wrong data is between 0.1% and 3.4%.^27^ Therefore, it is necessary to detect and eliminate the abnormal data before constructing our QSPR models. The Monte-Carlo (MC) cross-validation method has been proved to be an effective method for abnormal data detection.^26^ It has been successfully applied in the abnormal data detection of QSAR models.^28^ However, in the MC method, the threshold to eliminating the abnormal data is given by experience, so the rejected data cannot be guaranteed to be the most appropriate.

In this paper, the hybrid quantum particle swarm optimization (HQPSO) algorithm^29^ and 5-fold cross-validation technology were employed to determine the optimal threshold of the MC method. HQPSO was proposed in our study in 2019. It is a variant of quantum-behaved particle swarm optimization (QPSO). In HQPSO, new global, local and enhanced search strategy, Lévy flight and hopping operation technology, and new convergence speed control method were introduced. The performance of the algorithm is better than that of the traditional optimization algorithms such as GA, PSO and particle swarm optimization (QPSO), and it has been successfully applied to the optimization of the ground-state structure of the Au_*n*_ (*n* = 12–30) cluster (a typical NP problem) and hyper parameter selection of the QSAR model for acute toxicity on fathead minnow.^30^ The detailed implementation steps and mathematical equation of HQPSO algorithm can be found in the literature.^29^

The implementation steps of data filtering are as follows:

(1) Monte-Carlo cross-validation.

**Fig. 2:**
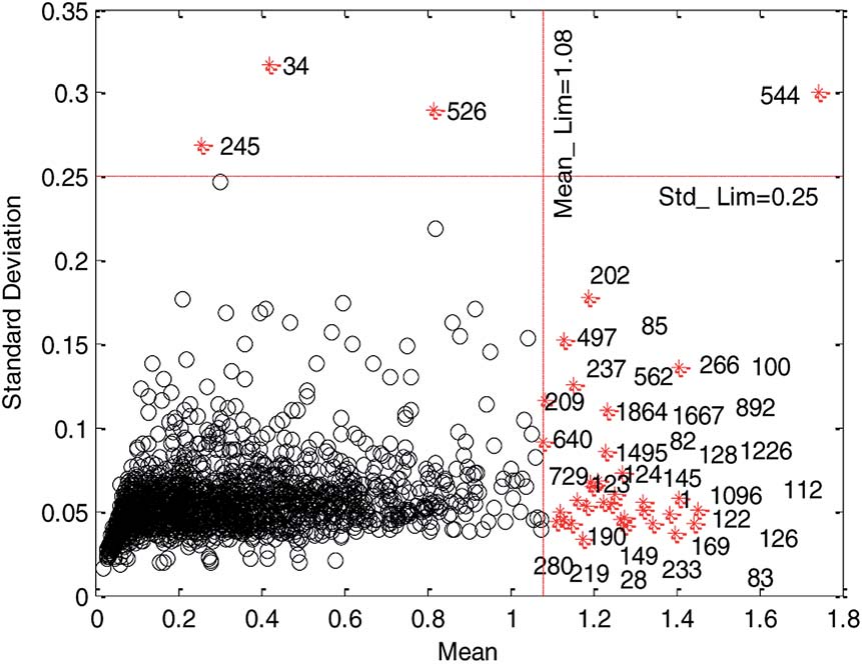
Mean of residuals *versus* standard deviation of residuals on initial modeling data set. (Black circles represent normal data and red cross stars represent abnormal data.)

In the MC method,^26^ the whole data were randomly divided into two parts, which are training set (90% of initial modeling data set) and test set (10% of initial modeling data set), respectively. The training set was used to establish the model using MLR. The test set was used to predict and the prediction error would be obtained for each test sample. This cycle was repeated 2000 times. Finally, the distribution of prediction error (mean and standard deviation of residuals) for each sample was obtained, and it is graphically shown in Fig. 2.

In Fig. 2, data to the right of the red vertical dashed line and above the red horizontal dashed line were considered as abnormal data and plotted as red cross stars. The black circles represent normal compounds in the data set. Only by reasonably determining the location of the two red dashed lines can the abnormal data in the data set be removed correctly.

(2) HQPSO optimization and 5-fold cross-validation.

We believe that a good data set should have good robustness, that is, high cross-validation accuracy. Therefore, we employ HQPSO to determine the optimal location of the two red dashed lines in Fig. 2, that is, the threshold recorded as Mean_Lim and Std_Lim. The parameters of the HQPSO algorithm are set as follows: the population size is 30, the number of maximum iterations is 2000, and the internal parameters are *λ* = 1 and *L* = 10 (the values of *λ* and *L* are selected according to ref. 29). The value of Mean_Lim ranges from 0.6 to 1.8, and the value of Std_Lim ranges from 0.1 to 0.35.

Primarily, for each iteration optimization, the values of Mean_Lim and Std_Lim were predetermined by HQPSO, and the abnormal data were removed according to Mean_Lim and Std_Lim. Subsequently, the reserved data were used to establish a 5-fold cross-validation model through MLR. Taking *R*_cv5_^2^ (the square correlation coefficients of 5-fold cross-validation) as the objective function, the best values of Mean_Lim and Std_Lim were selected corresponding to the maximum value of *R*_cv5_^2^. To avoid deleting too much data, thus reducing the diversity of modeling data set, we set a constraint condition, that is, the deleted data should not be more than 2% of the total data. When the iteration times reached 2000, the final optimization results were output as follows: Mean_Lim = 1.08, Std_Lim = 0.25.

According to the optimized threshold obtained by HQPSO, 37 outliers were eliminated. The reserved 1827 compounds were used to develop the QSPR models. The data set is listed in the ESI (QSPR_modeling_Data.xls).†

### 2.4. Data splitting

In the OECD principle,^31^ external validation is the only way to confirm the true predictive ability of a QSPR model. The real predictive ability of a QSPR model must be characterized by the predictive accuracy of the property of the compounds not used in the model development. This type of assessment requires the use of a test set. It can be seen from previous studies^32–34^ that the predictive ability of a QSAR model for different structural compounds will be better if diverse training data were obtained. To ensure the diversity of the training and test sets from structures and permeability values, we employed the idea of “clustering before classification”.

**Fig. 3:**
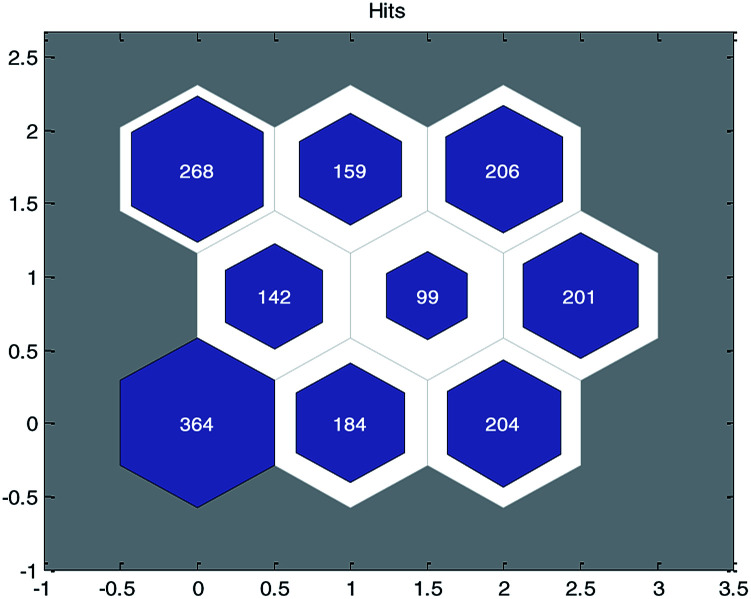
Clustering results of modeling data with self-organizing mapping (SOM) neural networks. (The number in each node represents the number of compounds contained in the node.)

First, the principal component analysis (PCA) method was used to deal with the input variables of modeling data set, and the first K principal components whose cumulative contribution rate was more than 90% (in this paper, *K* = 40) were selected as the input of SOM neural network. After that, the SOM divided the data set into 9 groups and each group of data had structural similarity. The clustering results are shown in Fig. 3.

Then, for each group of data, we sorted the data in ascending order according to their permeability values (log Papp) and picked one out of every five to constitute the test set. Finally, we obtained a training set containing 1458 compounds and a test set containing 369 compounds. A detailed classification is listed in the ESI (Training_and_Test_Set.xls).†

**Fig. 4:**
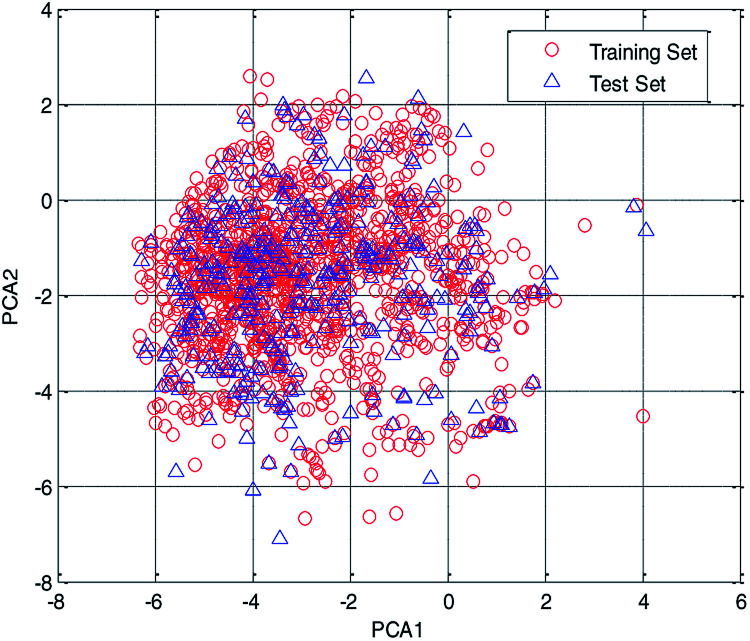
Chemical space analyzed using the principal component analysis (PCA) method.

In this study, the chemical space was analyzed using principal component analysis (PCA).^35–37^ As shown in the PCA plot of the compounds based on the 261 selected descriptors (Fig. 4), the compounds in the test set were basically distributed within the chemical space of the training set.

### 2.5. Feature selection

In QSPRs, using fewer descriptors helps to avoid over-fitting and to establish meaningful models, for which the chemical mechanisms are easy to explain. At the same time, deleting the unimportant descriptors will reduce the computational complexity. To select the key descriptors for our QSPR models, we implemented the following three operations:

(1) Descriptor importance evaluation by the MDI method.

MDI method has been successfully applied to evaluate the descriptor importance in our previous study.^30^ The basic idea is to scramble the values of each descriptor in turn and observe its influence on the model accuracy. Variables that have a significant impact on model accuracy are also of great importance. The detailed implementation process of the MDI method can be found in the literature.^30^ In this paper, MLR was employed to implement the regression model in MDI.

(2) Selection of a suitable set of descriptors.

**Fig. 5:**
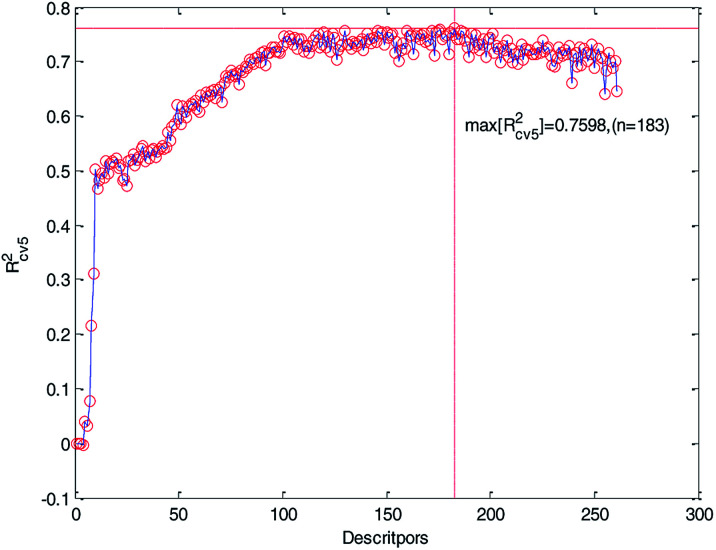
Relationship between *R*_cv5_^2^ and the number of descriptors (261 descriptors).

We ranked the descriptors in descending order according to their importance obtained by the MDI method and then gradually increased the number of descriptors in the training to construct MLR models. Meanwhile, the 5-fold cross-validation accuracy of each MLR model was recorded and plotted in Fig. 5. As can be seen from Fig. 5, *R*_cv5_^2^ has the maximum value when the number of descriptors is 183. At the same time, we can also see that too much descriptors will make the performance worse. Therefore, we chose the first 183 descriptors for further processing.

(3) Key descriptors selection with HQPSO.

In reported studies,^3,5,16,19–23^ the number of descriptors used in most Caco-2 permeability prediction models was less than 60. Therefore, 183 descriptors are still too many. In essence, the MDI method is a single-factor analysis method. Although the number of descriptors has been reduced, the selected descriptors may still have multicollinearity, which will affect the performance of the QSPR models. In this study, we used HQPSO combined with MLR to find the key descriptors, which benefits the predictive ability of the model. We took *R*_cv5_^2^, the 5-fold cross-validation accuracy of the MLR model established with the training data, as the optimization objective of HQPSO to select key descriptors. To avoid getting too many descriptors, we set a constraint that the number of descriptors does not exceed 60. The parameters of the HQPSO algorithm are set as follows: the population size is 30, the number of maximum iterations is 5000, and the internal parameters are *λ* = 1 and *L* = 10 (the values of *λ* and *L* are selected according to ref. 29).

**Fig. 6:**
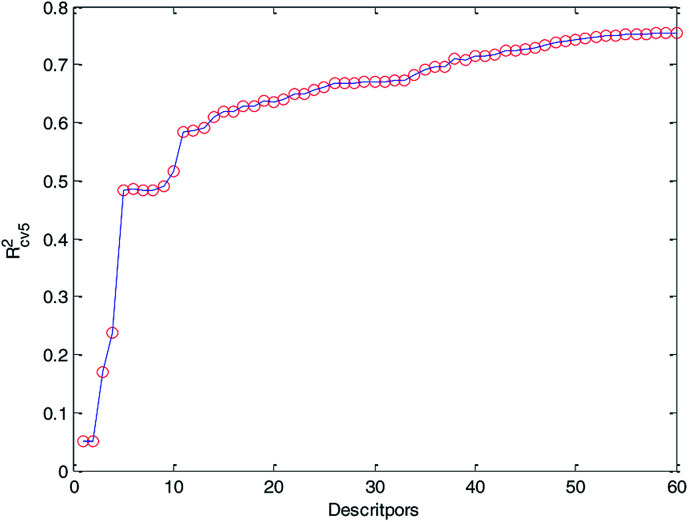
Relationship between *R*_cv5_^2^ and the number of descriptors (60 key descriptors).

Finally, we obtained 60 descriptors and repeated the operation (2) to the 60 descriptors, and the 5-fold cross-validation accuracy of each MLR model was recorded and it is plotted in Fig. 6. Compared with Fig. 5 and 6, *R*_cv5_^2^ of the MLR model with 60 optimized descriptors can basically reach the accuracy of the MLR model with 183 descriptors. Therefore, we can draw the conclusion that HQPSO is effective in selecting key descriptors.

At the same time, from Fig. 6, we can find that when the number of descriptors is greater than 50, more descriptors have little effect on improving the performance of the model. From the perspective of practical application, we prefer a simple predictive model. Finally, 50 descriptors were selected to develop our QSPR models. Details of the selected descriptors and their specific meanings are listed in the ESI (Table S1†).

### 2.6. Model building methods

In this paper, in order to get a superior QSPR model, we tried six methods and selected the best one for further research. The six methods are MLR, SVR, xgboost, RBF, dual-SVR and Dual-RBF. MLR multiple linear regression is the most popular method in QSAR/QSPR research.^38^ SVR, support vector machine regression, is an algorithm based on the structural risk minimization principle from statistical learning theory, and it is developed from the support vector machine (SVM) algorithm and mainly used in nonlinear regression analysis.^39^ Xgboost is an open-source machine learning project developed by Chen and his partners. It has effectively implemented the DGBT algorithm and made many improvements in algorithm and engineering.^40^ It has been widely used in the Kaggle competition and many other machine learning competitions and achieved good results. It is a very potential algorithm for QSAR modeling. RBF, radial basis function neural network,^41,42^ consists of three layers. The input layer is composed of some perceptual units, which connect the network with the external environment.

**Fig. 7:**
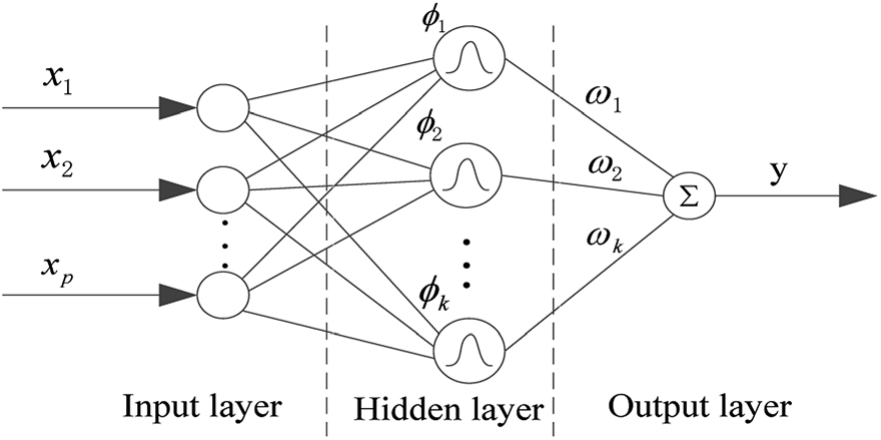
Structure of the RBF neural network.

The second layer is the only hidden layer in the network, and its function is to make a nonlinear mapping from the input space to the higher dimension hidden layer space; the output layer is linear, which provides response for the activation mode acting on the input layer. It has a powerful nonlinear fitting ability and a fast training speed. The structure of a network with *p* inputs, *k* hidden nodes, and 1 output is shown in Fig. 7.

The output of the network is as follows:1
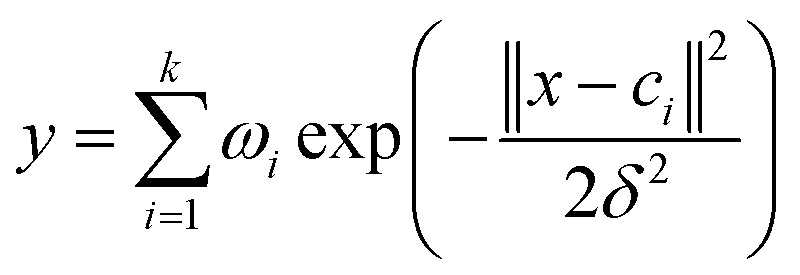
where *x* is the *p*-dimensional input vector, *c*_*i*_ is the center vector of the *i*th hidden layer node, *δ* is the spread of the radial basis function (activation function), and *ω*_*i*_ is the weight from the *i*th hidden layer node to the output node. The parameters affecting the performance of the RBF model are *δ* and *ε* (the mean squared error of the experimental and calculated responses of the training object. The smaller the value of *ε* is, the stronger the fitting ability of the trained model will be. However, too small *ε* will lead to over fitting of the model). *δ* and *ε* affect the fitting and generalization ability of the model, respectively.^30,43^

Dual-RBF is composed of two RBF neural networks in parallel, and the average output of the two networks is taken as the predicted output of the model. The idea of Dual-RBF comes from integrated learning. In order to obtain a superior QSPR model, we try to combine two RBF neural networks in parallel and integrate the two weak learners into a strong learner through the joint optimization of their hyper parameters (*δ* and *ε*) by HQPSO.

Dual-SVR, like dual-RBF, is composed of two SVR models in parallel, and the average output of the two networks is taken as the predicted output of the model.

### 2.7. Evaluation criterion

To select the best model from the above-mentioned six methods, we employed the following six parameters to make a preliminary evaluation of their performance: the square correlation coefficients of cross-validation (*R*_cv5_^2^), the root mean squared error of cross-validation (RMSE_cv_), the square correlation coefficients of fitting (*R*^2^), the root mean squared error of fitting (RMSE_F_), the square correlation coefficients of fitting for test set (*R*_T_^2^), and the root mean squared error for test set (RMSE_T_).^3^ The best QSPR model was selected by considering the above performance and running speed. Then, the Y-randomization test and the Golbraikh and Tropsha test were performed for further evaluations.

## 3. Results and discussion

### 3.1. Diversity comparison and analysis

(1) Compared with other literature.

**Fig. 8:**
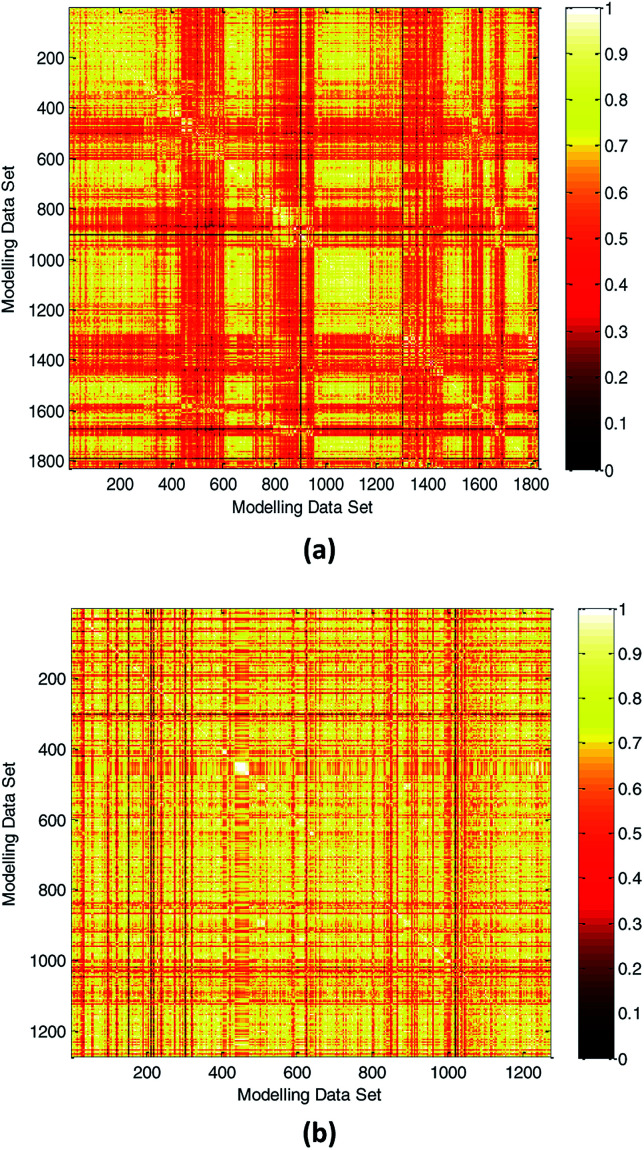
Chemical diversity distribution of modeling data set. (a) The normalized Tanimoto similarity index for all chemicals used in this study. (b) Normalized Tanimoto similarity index for all chemicals used in the literature.^3^ (The color closer to white in the heat map means that the compounds are more similar and the color closer to dark means that the compounds have higher diversity.)

In practical application, the high diversity of the modeling data is helpful to obtain a QSPR model with wide AD. Our modeling data set was obtained partly from the literature.^3^ To illustrate the diversity of our modeling data set, we compared the diversity with the literature.^3^ The Tanimoto similarity index was calculated using 50 selected descriptors to evaluate the chemical diversity of the compounds. A lower Tanimoto similarity index means a more diverse data set.^44,45^ The heat map of the normalized Tanimoto similarity index is shown in Fig. 8(a). The Tanimoto similarity index of the data set used in the literature^3^ was also calculated, and the normalized Tanimoto similarity index is shown in Fig. 8(b). The average similarity between two compounds in this study and literature^3^ was 0.6098 and 0.6941, respectively. Therefore, we obtained a more diverse data set, which helps to expand the scope of application of the model.

(2) Compared with the training set and test set.

**Fig. 9:**
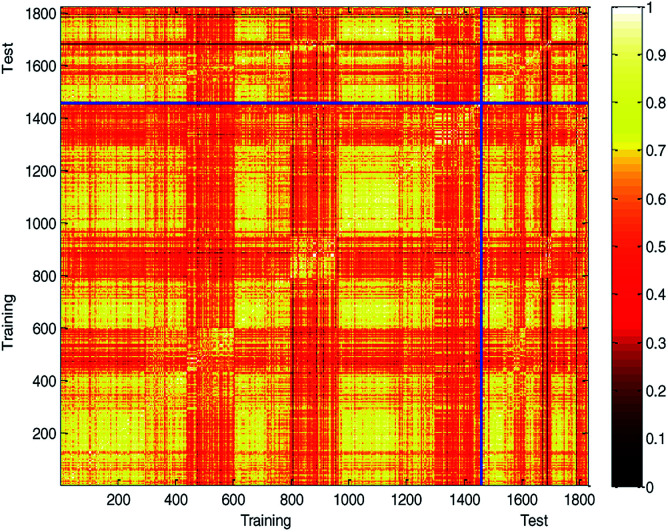
Comparison of chemical diversity between the training and test sets.

Generating multiple chemically diverse training and test sets is helpful to establish a stable QSPR model and obtain real model accuracy statistical data. The Tanimoto similarity index of training set, test set and training set between test set were also calculated to evaluate the chemical diversity of the compounds in the training set and test set. The normalized Tanimoto similarity index of the training and test sets is shown in Fig. 9.

The average similarity between two compounds in training set, test set, and training set between test set was 0.6109, 0.6060, and 0.6082, respectively. The similar Tanimoto similarity index illustrates that they have similar chemical diversities.

**Fig. 10:**
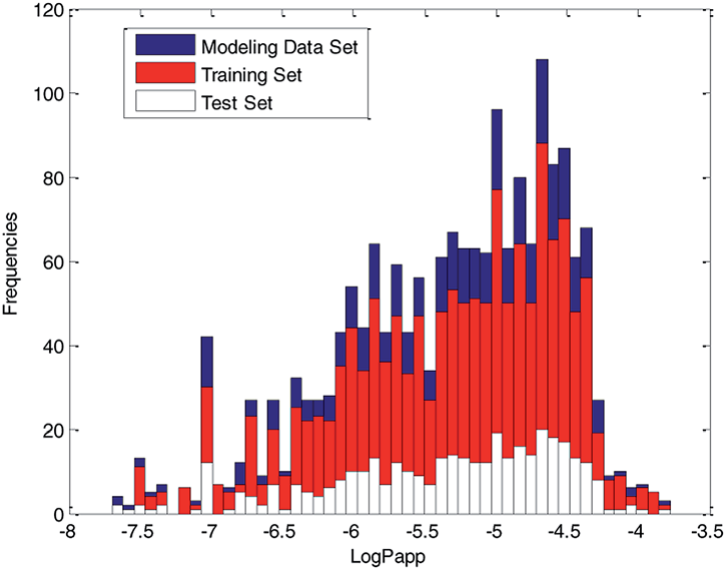
Distribution of log Papp values in modeling data set, training set and test set.

For further analyses, the distribution of log Papp values was analyzed using a histogram, which is shown in Fig. 10. From Fig. 10, we can see that the ratio of various log Papp values in the training and test sets are basically uniform, about 4 : 1. Therefore, we conclude that the data splitting method ensured the symmetry and fairness of the data splitting.

### 3.2. Model building and selection

We generated our QSPR models using MLR, RBF, SVR, xgboost, dual-SVR and dual-RBF with the 50 selected descriptors, respectively. To ensure the performance of these models, except for MLR, the hyper parameters of other 5 models were optimized by HQPSO.

For the above-mentioned algorithms, MLR has no hyper parameters. Xgboost is a boosting algorithm, which is not easy to over fitting. Therefore, for the xgbobost model, we need to consider only the robustness when determining its hyper parameters. The fitness function of the HQPSO algorithm used for xgboost is defined as eqn (2):2
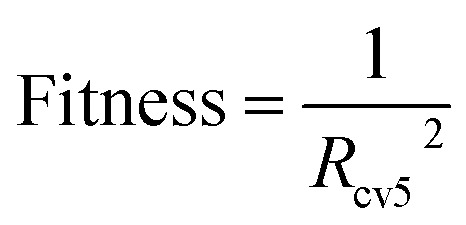


RBF and SVR are nonlinear models, and although RBF has strong fitting ability, it is easy to over fitting. During the experiment, we found that the value of *R*^2^ is too large, which will cause the value of *R*_cv5_^2^ to become smaller. The value of *R*_cv5_^2^ is too large, which also causes the value of *R*^2^ to be smaller (the values of *R*^2^ and *R*_cv5_^2^ were adjusted by the hyper parameters of RBF).^30^ That is, if the fitting ability of the model is too strong, the robustness of the model will be weaker, and if the robustness of the model is too strong, the fitting ability of the model will be weaker. Only choosing the reciprocal of *R*^2^ or *R*_cv5_^2^ as the fitness cannot make the model get good performance. Like RBF, SVR has the same problem. Therefore, for RBF and SVR, to balance the fitting ability and robustness, the fitness function of the HQPSO algorithm is defined as eqn (3):3
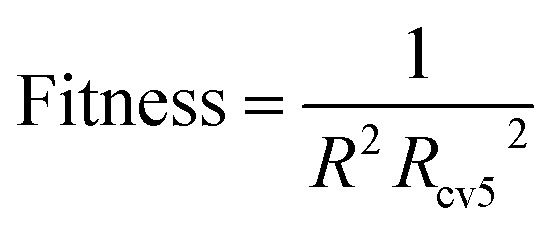


Dual-RBF and dual-SVR are integrated models of RBF and SVR, respectively. They also have over fitting problems. Therefore, the fitness function of the HQPSO algorithm used for dual-RBF and dual-SVR is also defined as eqn (3). When HQPSO was used for hyper parameter optimization, the parameters of the HQPSO algorithm were set as follows: the population size was 30, the number of maximum iterations was 1000, and the internal parameters were *λ* = 1 and *L* = 10 (the values of *λ* and *L* were selected according to ref. 29). To avoid the illegal value of fitness, when the value of *R*^2^ or *R*_cv5_^2^ is not greater than 0, we set their values to 0.0001.

The performance and the hyper parameters of the six models are shown in Table 1. Tropsha *et al.* suggested that the available QSAR model should satisfy *R*^2^ > 0.6 and *R*_cv5_^2^ > 0.5.^46^ From Table 1, we can see that the models all have relatively good performance (*R*^2^ > 0.7, *R*_cv5_^2^ > 0.7, and *R*_T_^2^ > 0.7), which indicate that the selected descriptors can effectively predict Caco-2 permeability values. In the table, MLR is a linear model, and it has the worst fitting ability (*R*^2^ = 0.76) and external prediction ability (*R*_T_^2^ = 0.70), which indicates that there may be a nonlinear relationship between the selected descriptors and the Caco-2 permeability values. Therefore, nonlinear models are suitable for predicting Caco-2 permeability values. Among the six models, xgboost has the best fitting ability (*R*^2^ = 0.99), and dual-SVR and dual-RBF have the best external predictive ability (*R*_T_^2^ = 0.76, RMSE_T_ = 0.39). Although xgboost has the best fitting ability, its robustness and external predictive ability are all weaker than that of dual-SVR and dual-RBF. If we do not consider the running speed of the algorithm, judging from the performance of the six QSPR models on the whole, we think dual-SVR is the best one, and the performance of dual-RBF is similar to that of dual-SVR. The fitting ability of dual-RBF is slightly worse than that of dual-SVR, but they have the same robustness and external predictive ability. From Table 1, we can also see that the performance of dual-RBF and dual-SVR is better than that of RBF and SVR, respectively.

**Table 1:** The parameters and performance of the six QSPR models

Method	Performance						Hyper parameters
	*R* ^2^	RMSE_F_	*R*_cv5_^2^	RMSE_cv_	*R*_T_^2^	RMSE_T_	
MLR	0.76	0.38	0.74	0.39	0.70	0.44	—
SVR	0.92	0.22	0.74	0.39	0.74	0.41	C = 3, nu = 0.8027, width = 2.0239
RBF	0.90	0.24	0.73	0.41	0.72	0.42	*δ*_1_ = 2.0545, *ε*_1_ = 0.0155
Xgboost	0.99	0.07	0.74	0.39	0.74	0.41	learning_rate = 0.05, max_depth = 6, n_estimators = 600
Dual-SVR	0.92	0.22	0.77	0.37	0.76	0.39	C_1_ = 3, *ν*_1_ = 0.3106, width_1_ = 1.2824
							C_2_ = 49, *ν*_2_ = 0.4662, width_2_ = 12.1833
Dual-RBF	0.91	0.24	0.77	0.37	0.76	0.39	*δ*_1_ = 6.5400 *ε*_1_ = 0.0296
							*δ*_2_ = 1.7100 *ε*_2_ = 0.0101

**Fig. 11:**
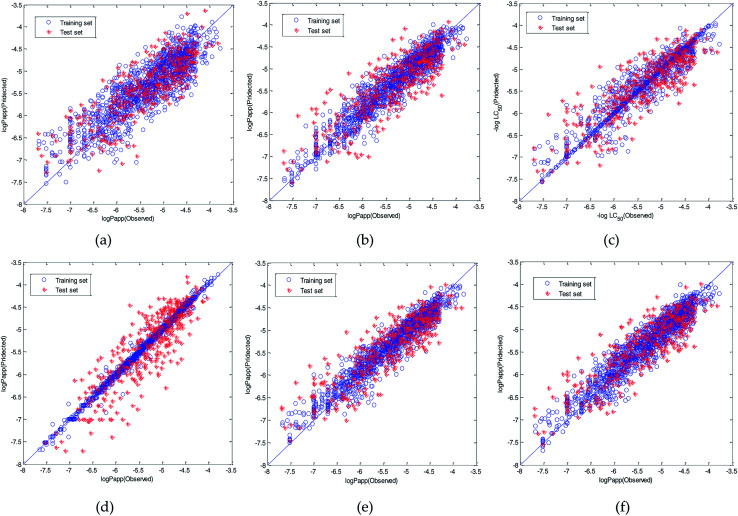
Visual predictive performance of the six quantitative structure-property relationship (QSPR) models. (a) Multivariate linear regression (MLR). (b) Radial basis function (RBF) neural network. (c) Support vector machine regression (SVR). (d) Xgboost. (e) Dual-SVR. (f) Dual-RBF. (The blue circles represent the compounds in the training set, and the red cross stars represent the compounds in the test set. The blue solid line shows that the experimental and predicted values are the same.)

Therefore, the modeling strategy for QSPR constructing two weak learners into one strong learner through optimization algorithm is reasonable. The visual predictive performance of the six models is shown in Fig. 11. Blue circles and red cross stars are distributed more-or-less symmetrically on both sides of the solid line, which indicates the good predictive ability of the models.

Our purpose is to establish a QSPR model with superior performance and high speed suitable for virtual screening. We further investigated the run speed of dual-RBF and dual-SVR in predicting the Caco-2 permeability of compounds. When the two models were applied to predict the test set (369 compounds), we run their Matlab code 100 times and recorded their run time (Software version: Matlab2018, Operating system: Windows 10, CPU: Inter(R) Core(TM) U5-9400 CPU@2.9GHZ, RAM:8.00G). Then, the average run time was calculated. The average run time of dual-RBF and dual-SVR was 0.42 s and 0.71 s, respectively. In terms of running speed, dual-RBF is obviously faster than dual-SVR, and hence, it is more suitable for the virtual screening of compounds. Therefore, we choose dual-RBF as the best model for further research.

In the OECD principle, the validation of the QSPR model becomes an essential step in developing a statistically valid predictive model. Therefore, a normative QSPR model in line with OECD principles must be comprehensively verified.

**Fig. 12:**
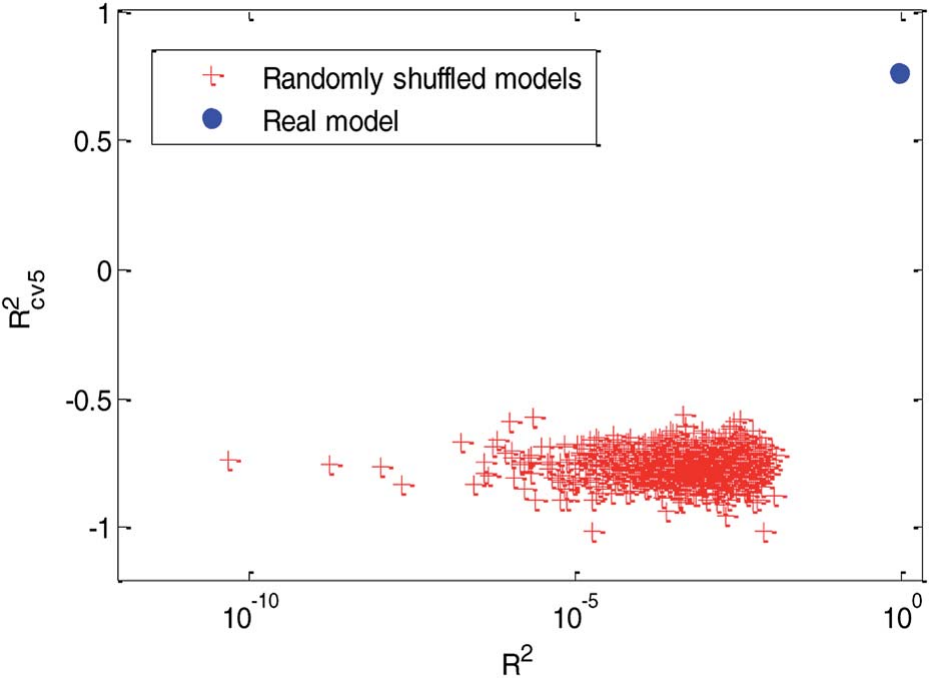
Distribution of *R*^2^ and *R*_cv5_^2^ of randomly shuffled models in the Y-randomization test compared with the real model. (The solid blue circle represents the performance of the real QSPR model established with the training set, and the red cross stars represent the performance of randomly shuffled models in Y-randomization tests.)

In addition to the above-mentioned basic validation indexes, the dual-RBF model was evaluated more strictly, as follows:

(1) Over fitting: *R*^2^ − *R*_cv5_^2^ = 0.91–0.77 = 0.14 < 0.3, and *R*_T_^2^ is slight lower than *R*_cv5_^2^, indicating that the model can avoid the over fitting phenomenon, and the established model is reliable and generally applicable.^47^

(2) Topliss ratio: our QSPR model also fulfills the rule of thumb condition (that is, the Topliss ratio), whereby the chemical number in the training set over the number of selected variables should be at least 5 (1458/50 = 29.16 > 5).^48^

(3) Y-randomization test: in this test, the dependent-variable vector (Y-vector) was randomly shuffled, and a new QSPR model was developed using the original independent variable matrix. It was expected that the resulting QSPR models would have low *R*^2^ and *R*_cv5_^2^ values.^49^ This process was repeated 1000 times, and the results for 1000 runs are graphically shown in Fig. 12.

From Fig. 12, we can see that the red cross stars are far from the solid blue circle. It indicated that the performance of the real model is quite different from the randomly shuffled models. The high quality of *R*^2^ (*R*^2^ = 0.91) indicate that the real model has a good fitting ability. The relatively high value of *R*_cv5_^2^(*R*_cv5_^2^ = 0.77) in the cross-validation test and the poor values of *R*^2^ (range from 4.752 × 10^−11^ to 0.012) and *R*_cv5_^2^ (range from −1.0166 to −0.5639) in the Y-randomization tests ensured the robustness of the model. The poor performance of these randomly shuffled models suggest that our QSPR model established by dual-RBF also reflects the true relationship between the selected molecular descriptors and Caco-2 permeability values rather than chance correlation.^3^

(4) Golbraikh and Tropsha criteria^50^ were also used to evaluate the performance of the dual-RBF model. *K* = 1.0014(*k*′ = 0.9934) and (*R*_0ext_^2^ − *R*′_0ext_^2^)/*R*_0ext_^2^ = 0.0019, where *K* and *k*′ are the corresponding slopes of regression lines through the origin. *R*_0ext_^2^ and *R*′_0ext_^2^ were calculated forcing the regression line to pass through the origin. For acceptable QSPR predictive models, 0.85 < *k*, *k*′ < 1.15 and (*R*_0ext_^2^ − *R*′_0ext_^2^)/*R*_0ext_^2^ < 0.1.^51,52^

In conclusion, the dual-RBF model passed the validation successfully. It is indeed a statistically valid QSPR model.

### 3.3. Mechanism interpretation

OECD principles suggest that the mechanism of a QSAR/QSPR model should be explained, if possible. The QSPR model using different combinations of descriptors may have similar predictive performance, and it is difficult to explore its clear mechanism, but some useful hints can always be found in the model, which may be useful to medicinal chemists.

It is generally believed that the permeation of a compound is a complex process influenced by a different kind of interaction. Generally, a shaped or size-suitable compound with proper polarity and lipophilicity is considered to have a better Papp value and can effectively permeate the cell membrane.^3^ As has been mentioned in previous studies,^3,53–55^ the hydrogen bond donor, surface area or molecular size/weight, polarizability, charge, *etc.* have a more or less effect on the permeability. It can be clearly seen from the ESI (Table S1†) that there are ten descriptors related to hydrogen bonds, two descriptors related to the molecular size, three descriptors related to the charge and five descriptors related to the polarizability. This indicates that although different descriptor computing software was used, the descriptors selected in our research are consistent with previous studies to a great extent. We can also find that some new type of descriptors were selected in our research such as descriptors related to “H E-state”, ETA_Epslion2 (a measure of electronegative atom count), and C3SP3 (singly bound carbon bound to three other carbons).

**Fig. 13:**
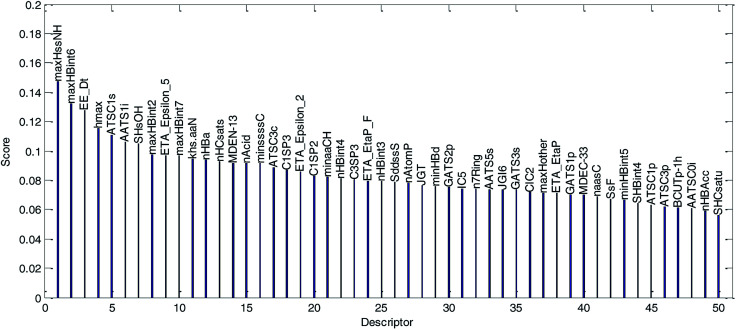
Variable importance was evaluated by the MDI method and sorted in descending order. (The descriptor with the best score is the most important variable.)

To compare every molecular descriptor and interpret some important descriptors effectively, we performed the following preparation steps:

(1) The variable importance of each selected descriptor was evaluated by the MDI method. The dual-RBF model has been considered as the best one of the six established QSPR models, and hence, the dual-RBF model with optimized hyper parameters listed in Table 1 was employed to implement the regression model in MDI. The evaluation results of variable importance are shown in Fig. 13.

(2) To explore further the relationship between Caco-2 permeability and important descriptors, the correlations between each of the top ten important descriptors and log Papp were calculated and they are listed in Table 2, and the other descriptors' obvious correlations with Papp values (*R* > 0.5 or *R* < −0.5) are also collected in Table 2.

**Table 2:** Information of the top 10 important descriptors' and other descriptors' obvious correlations with log Papp values

Importance ranking	Descriptor name	Correlation	Description
1	maxHssNH	−0.35	Maximum atom-type H E-state: –NH–
2	maxHbint6	−0.51	Maximum E-State descriptors of strength for potential hydrogen bonds of path length 6
3	EE_Dt	−0.42	Estrada-like index from detour matrix
4	hmax	−0.3	Maximum H E-state
5	ATSC1s	0.42	Centered Broto-Moreau autocorrelation − lag 1/weighted by I-state
6	AAS1i	−0.25	Average Broto-Moreau autocorrelation − lag 1/weighted by first ionization potential
7	SHsOH	−0.35	Sum of atom-type H E-State: –OH
8	maxHBint2	−0.39	Maximum E-state descriptors of strength for potential hydrogen bonds of path length 2
9	ETA_Epslion_5	−0.02	A measure of electronegative atom count
10	maxHBint7	−0.49	Maximum E-state descriptors of strength for potential hydrogen bonds of path length 7
12	nHBa	−0.56	Count of E-states for (strong) hydrogen bond acceptors

We explained the permeability mechanisms combined with the importance of the descriptors (Fig. 13) and the correlations between the descriptors and log Papp values (Table 2). Then, we made the following inferences:

(1) “maxHssNH” is the most important descriptor affecting permeability in the dual-RBF model and the correlation coefficient is −0.35. The descriptor is related to the “H E-state” of “–HN–” fragments in compounds. Although the correlation between “maxHssNH”” and log Papp is not significant, it can still reflect the change trend of the log Papp value to a certain extent. As far as we know, this is a new descriptor and has not been used in Caco-2 permeability studies as an important descriptor. As important descriptors, “hmax” and “SHsOH” are also related to the “H E-state”. This indicated that the “H E-state” may be an important factor affecting permeability.

(2) “maxHbint6”, “maxHBint2”, “maxHbint7” and “nHBa” are related to hydrogen bonds. “maxHbint6” and “nHBa” have obvious correlations with log Papp (the correlation coefficient is −0.51 and −0.56, respectively). Hydrogen bonding has been identified as an important parameter for describing drug permeability.^3,53,54^ As the values of these descriptors increase, the permeability will be weaker. They can reflect the changing trend of permeability of compound to some extent.

(3) “EE_Dt” is a topological descriptor related to “Estrada-like index from detour matrix”. Although its physical meaning is not clear, the topological structure of molecules does affect the permeability.

(4) “ATSC1s” is an autocorrelation descriptor. It is the only descriptor with an obvious positive correlation with log Papp in Table 2 (The correlation coefficient is 0.42). Although its physical meaning is not clear, it plays an active role in reflecting the change trend of permeability.

(5) “AAS1i” is also an autocorrelation descriptor. The value of “AAS1i” is weighted by the first ionization potential. The first ionization potential is the energy required for a gaseous atom in the ground state to lose one electron in its outermost layer. The larger the initial ionization energy, the harder it is for an atom to lose an electron. “AAS1i” has no obvious correlation with log Papp (the correlation coefficient is −0.25). Although it is an important descriptor in the dual-RBF model, it has little effect on indicating the permeability. There may be certain nonlinear relationship between it and permeability.

(6) “ETA_Epslion_5” is related to electronegative atom counts. It is a relatively important descriptor, but has little correlation with log Papp. This indicates that there is some nonlinear relationship between them, and we cannot use it to indicate the change trend of permeability.

In a word, the permeation mechanism of compounds through Caco-2 cell is very complex and cannot be described by several descriptors. Although the hydrogen bond donor and the “H E-state” are essential factors affecting the permeability, there is a complex nonlinear relationship between the descriptors and permeability. Therefore, it is necessary to establish a permeability prediction model by using nonlinear tools, such as SVR or neural networks.

### 3.4. Applicability domain of the dual-RBF model

In the OECD principle, a standard QSAR model must give a defined domain of application. The AD indicates the applicable scope of a QSAR model. The AD evaluation is a guarantee for QSPR models in accurately and reasonably predicting newly synthesized compounds.^56^

Leverage^57^ is a representative and widely used distance-based AD definition method. It is essentially a method based on the spatial distance information between compounds in the training set, but it does not consider the importance of each descriptor.

In this paper, the importance of each descriptor used in the dual-RBF model has been evaluated, which shows that different descriptors have different effects on the proposed model. Therefore, considering the importance of selected descriptors, we proposed an IWD method to define the AD for the dual-RBF model and compared with the leverage method. The definition and implementation step of the IWD method are as follows:

Step 1: the weighted operation for each compound is performed: *v*_*ij*_ = *A*_*ij*_Score_*j*_. *A*_*ij*_ is the value of the *j*th descriptor of the *i*th compound, Score_*j*_ is the score of the *j*th descriptor calculated by the MDI method, as shown in Fig. 13, and *v*_*ij*_ is the weighted value of the *j*th descriptor of the *i*th compound in the training set.

Step 2: calculate the center point *C* = [*c*_1_, *c*_2_, …, *c*_*s*_], and *c*_*j*_ is written in eqn (4):4
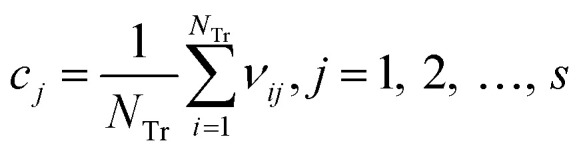
where *N*_Tr_ is the number of compounds in the training set.

Step 3: calculate the Euclidean distance of each weighted compound to the center *C* using eqn (5).

5 d_*i*_ = ‖*v*_*i*_ − *C*‖, *i* = 1, 2,…, *N*_Tr_

Step 4: calculate the mean value and standard deviation of *d.* The mean value and standard deviation are expressed as *u* and *δ*, respectively. For the *i*th compound, if *d*_*i*_ > *u* + 3*δ*, we consider the compound outside AD. Otherwise, it is inside AD.

**Fig. 14:**
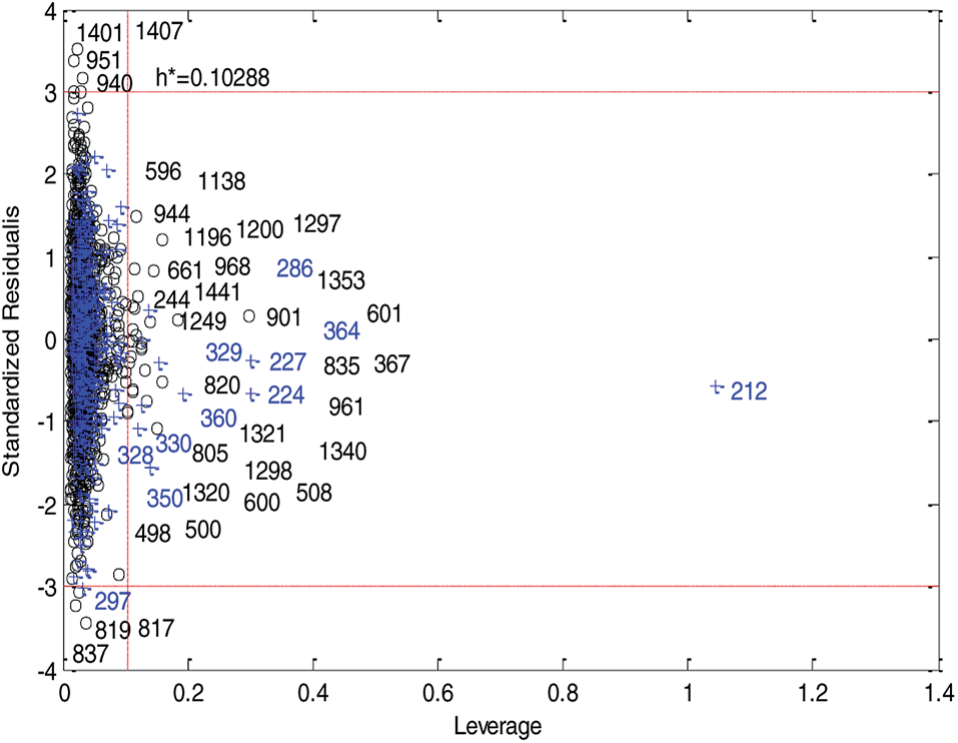
Williams plot of the consensus model based on the leverage method. (Black circles represent compounds in the training set, and blue crosses represent compounds in the test set.)

**Fig. 15:**
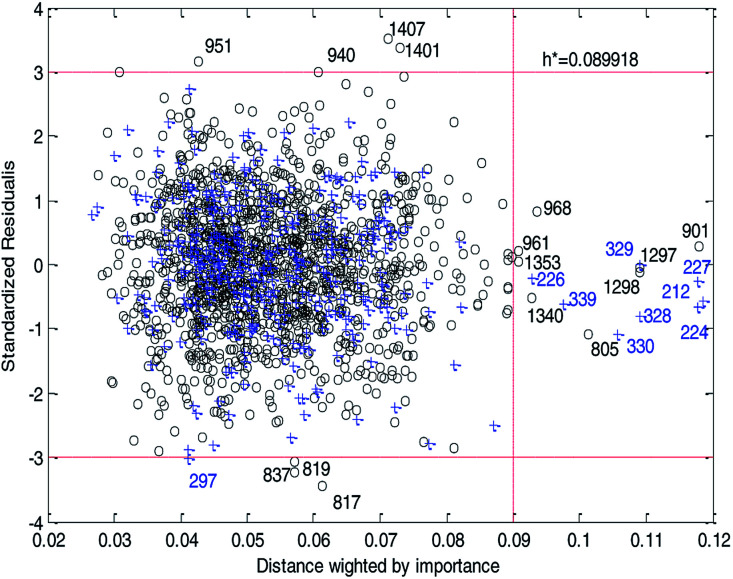
Williams plot of the dual-RBF model based on the IWD method. (Black circles represent compounds in the training set, and blue crosses represent compounds in the test set.)

Fig. 14 and 15 show the difference between the leverage and IWD methods. In Fig. 14 and 15 the transverse dashed lines represent a ±3 standard residual. In Fig. 14, the vertical dashed line represents a warning leverage, h* = 0.1029. In Fig. 15, the vertical dashed line represents a warning IWD, h* = 0.0899. It can be seen from Fig. 14 and 15 that only one compound in the test set was predicted outside the ±3 standardized residuals, which illustrates the good predictive ability of the dual-RBF model.

Comparing Fig. 14 with Fig. 15, we can clearly see that, regardless of the AD definition method, the outliers were all in their respective AD. Few outliers in training and test sets show that the MC method in Section 2.3 can effectively remove abnormal data in the modeling set. In the IWD method, the AD coverage rate of the dual-RBF model is 99.45% (98.15% in leverage method) for the training set and 97.83% (97.29% in leverage method) for the test set. The application scope of the model has been expanded. We delete compounds outside the AD in the leverage and IWD methods respectively, and recalculate *R*_T_^2^ and RMSE_T_. The new *R*_T_^2^ = 0.75, RMSE_T_ = 0.39 in the leverage method, and new *R*_T_^2^ = 0.74, RMSE_T_ = 0.39 in the IWD method. Therefore, the expansion of the AD in the IWD method did not destroy the performance of the dual-RBF model obviously. From the performance of the IWD method, the AD definition method based on IWD is reasonable.

From the two above-mentioned domain definition methods, we can see that the dual-RBF model established in this paper has a wide AD.

### 3.5. Outlier analysis of the dual-RBF model

**Fig. 16:**
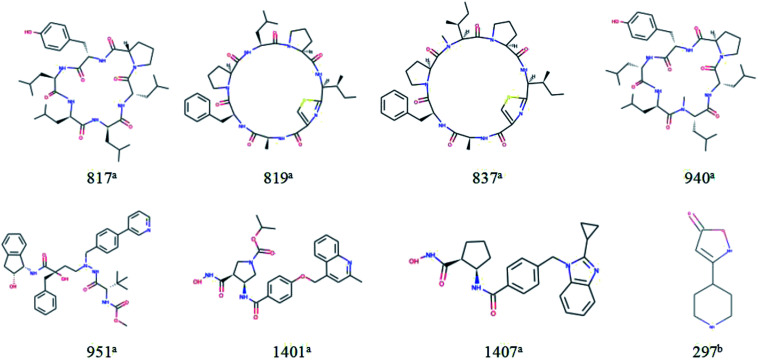
Molecular structures of the outliers. ^a^Outliers in the training set; ^b^outliers in the test set.

For response variables, 8 compounds were identified as outliers because their standardized residuals were outside the range of ±3 standardized residuals, as shown in Fig. 15. The molecular structures of the outliers are shown in Fig. 16. The detailed information for these outliers and four important descriptors that have obvious correlation with log Papp are listed in Table 3.

**Table 3:** Information on the outliers in the dual-RBF model

No.	CAS or ChemBL	Experiment	Predict	*M*_W_	maxHbint6 *R* = −0.51	EE_Dt *R* = −0.42	ATSC1s *R* = +0.42	nHBa *R* = −0.56
817^a^	CHEMBL3427786	−6.8539	−6.0352	712.93	3.6324	709.7827	−9.5150	13
819^a^	CHEMBL3616777	−6.6990	−5.9318	721.92	5.0184	709.7827	−4.4795	13
837^a^	CHEMBL3616783	−6.3979	−5.6671	735.95	4.9984	709.7827	−4.1317	13
940^a^	CHEMBL3427789	−5.1062	−5.8193	726.96	3.6047	709.7827	−9.2161	13
951^a^	CHEMBL583371	−4.5850	−5.3381	693.85	4.4860	591.003	−13.8958	10
1401^a^	CHEMBL256598	−5.7696	−6.5720	506.56	8.8025	446.434	−6.3583	9
1407^a^	CHEMBL252470	−5.6990	−6.5329	418.50	8.3255	353.036	0.2125	6
297^b^	CHEMBL3287845	−6.3010	−5.1109	168.20	1.4294	65.8150	−0.1271	3

a Outliers in the training set.

b Outliers in the test set; *M*_W_: molecular weight; *R*: correlation coefficient; nHBa ranges from 0 to 37, ATSC1s ranges from −64.4300 to 7.6843, EE_Dt ranges from 10.7425 to 709.7827, and maxHbint6 ranges from 0 to 10.2700.

Combine the information in Fig. 15 and Table 3, we make the following inferences about the causes of outliers:

For 817^a^, 819^a^, 837^a^, and 940^a^, interestingly, these molecules all have relatively large molecular weight (*M*_W_ (molecular weight) > 700) and a complex ring structure. The reason for their large prediction errors maybe that there are few samples close to their molecular weight and structural characteristics in the training set, and the model does not learn their permeation mechanism well. For 297^a^, its molecular weight is smaller and its structure is simpler than that of most molecules in the modeling set. It has the same reason for its large prediction error. Therefore, to better predict the permeability of each compound, we need to use a larger number of compounds with representative structures as a training set to build a better QSPR model.

For 1401^a^ and 1407^a^, the values of “maxHbint6” are fairly large, which are 8.8025 and 8.3255, respectively. In the dual-RBF model, “maxHbint6” is the second important descriptor, and has an obvious negative correlation with log Papp. This may be the main reason for the predicted result of the model being smaller than the experimental value.

For 951^a^, no clear reason for the large prediction error is found in Table 3 except that the molecular weight is relatively large. From Fig. 16, we can find some stereoscopic structures on 951^a^, but in this paper, in order to ensure the stability of the model, we only use the 0–2D descriptors to characterize the molecular structure characteristics. This may be the reason for its large prediction error.

### 3.6. Comparison of the dual-RBF model with other published models

To better illustrate the superiority of the dual-RBF model established in this paper, we developed a detailed comparative analysis between the dual-RBF model and the main published regression models. Our interest was only on models established with a modeling set *N*_m_ > 200, because models with small data sets make it difficult to collect sufficient and diverse molecular information to develop a superior QSPR model with a wide AD. The results of this comparison can be found in Table 4.

**Table 4:** Comparison of the current model with previous models (*N*_m_ > 200)

Method	*N*_m_	*N*_de_	*R* ^2^	*R*_cv5_^2^	*R*_T_^2^	AD	Reference
GA-NN^a^	207	9	0.86	—	0.79	No	58
ANN^b^	296	12	0.84	—	0.77	No	17
Boosting	1272	30	0.97	0.83	0.81	Yes	3
RF^c^	15 791	8	0.52	—	—	No	15
Dual-RBF	1827	50	0.91	0.77	0.76	Yes	Present

a Genetic algorithm-neural network.

b Artificial neural networks.

c Random forest; *N*_m_ is the number of compounds in the modeling set, and *N*_de_ is the number of descriptors used in each model.

In terms of the normative nature of the modeling process, only the dual-RBF model and the boosting model in ref. 3 strictly follow the OECD principle. Although the number of descriptors used in the dual-RBF model is greater than that of the boosting, it satisfies the “Topliss ratio” condition, and there is no over-fitting. Meanwhile, the importance of the descriptors and the permeation mechanisms are explained reasonably. The other models have some disadvantages. GA-NN^58^ and ANN^17^ do not report their AD and they all have small modeling data set, which limits their applications. Although the RF model has the largest modeling data set compared with other models, its fitting ability is poor (*R*^2^ = 0.52), and it has not been cross-verified and externally verified. Therefore, the robustness and external prediction ability of the model cannot be guaranteed. Strictly speaking, it is not an available QSPR model.

By comparing the boosting model in ref. 3 and the dual-RBF model, we can see from Table 4 that the performance of dual-RBF model is worse than that of the boosting model. It seems that the model we established has no advantages. However, we cannot ignore the important issue that the difference in performance comes mainly from the modeling data set. The number of compounds used in the dual-RBF model was 1.5 times that of the boosting model. Commonly, for regression models, the more molecules the data set includes, the accuracy will be more difficult to improve and need more descriptors to build a reasonable model.^3^ To better illustrate the superiority of the dual-RBF modeling method, we established another dual-RBF model using the modeling data in the literature.^3^ The comparison results are listed in Table 5.

**Table 5:** Comparison results of boosting and dual-RBF model using the modeling data in the literature.^3^

Method	*R* ^2^	*R*_cv5_^2^	*R*_T_^2^	Hyper parameters
Boosting	0.97	0.83	0.81	—
Dual-RBF	0.90	0.83	0.83	*δ*_1_ = 1.4700 *ε*_1_ = 0.0090
				*δ*_2_ = 11.1000 *ε*_2_ = 0.0249

From Table 5, we can see that the fitting ability (*R*^2^) of boosting is better than that of the dual-RBF model, but the external prediction ability (*R*_T_^2^) of the dual RBF model is better than the boosting model. For the QSPR model, robustness (it is characterized by *R*_cv5_^2^) and external prediction ability are our primary consideration. Therefore, the performance of dual-RBF model is better than boosting, at least not worse than boosting. Since the boosting model in the literature^3^ did not give explicit hyper parameters, we cannot use the modeling data of this paper for comparative study. However, xgboost used in this paper is an optimized variant of the boosting method. For the modeling data in this paper, the performance of the dual-RBF model is obviously better than that of xgboost. Moreover, boosting inherits many sub-models, and its running speed is slow. Therefore, we can infer that the dual-RBF modeling method is more suitable for our study.

At the same time, from the comparison of diversity in Section 3.1, we can clearly see that the modeling data of the dual-RBF model is more diverse than that of boosting,^3^ which helps to expand the scope of application of the model. Meanwhile, different from boosting model, the dual-RBF model in this study only uses 0–2D descriptors, which greatly simplifies the descriptor calculation process and facilitates the rapid application of the model.

## 4. Conclusions

In this study, we have developed a new Caco-2 permeability prediction model based on dual-RBF neural networks under the OECD principle. A 0–2D descriptor was used to establish the QSPR model that can facilitate the rapid application of the model. The MC method combined with the HQPSO algorithm effectively removed the abnormal data in the modeling data. Through the SOM neural network and PCA method, the modeling data set was successfully split into training and test sets with multiple chemically diversity. Through the MDI method and HQPSO, 50 key descriptors were selected for QSPR modeling. The dual-RBF model with good performance and high speed was chosen as the best model from six models established from different methods. Combined with the importance of the descriptors evaluated by the MDI method, as well as the correlation between the descriptors and Caco-2 permeability, we explained the penetration mechanism and concluded that the “H E-state” and hydrogen bond donor are important factors affecting the permeability of Caco-2 cells in the dual-RBF model. A new IWD-based AD was defined, and the result showed that the QSPR model established in our study has a wide AD. The comparison results indicated that the dual-RBF model exhibits certain advantage in database size, data diversity, transparency of modeling process and prediction accuracy to some extent. As far as we know, the dual-RBF modeling method has not been used for the QSPR study. Under the OECD principle, this is the best QSPR model for Caco-2 permeability prediction with such a large data size. It will be a promising tool for virtual screening in the early stage of drug development.

## Conflicts of interest

The authors declare no conflict of interest.

## Supplementary Material

RA-010-D0RA08209K-s001
